# Music Therapy Alleviates Motor Dysfunction in Rats With Focal Cerebral Ischemia–Reperfusion Injury by Regulating BDNF Expression

**DOI:** 10.3389/fneur.2021.666311

**Published:** 2021-06-28

**Authors:** Weiguan Chen, Jiaxuan Zheng, Guangyu Shen, Xin Ji, Linlin Sun, Xia Li, Feng Xu, Jin-hua Gu

**Affiliations:** ^1^Department of Rehabilitation Medicine, Affiliated Hospital of Nantong University, Nantong, China; ^2^Department of Rehabilitation Medicine, The First Affiliated Hospital, Sun Yat-sen University, Guangzhou, China; ^3^Department of Clinical Pharmacy, Affiliated Maternity & Child Healthcare Hospital of Nantong University, Nantong, China; ^4^Department of Neurosurgery, Huashan Hospital, Shanghai Medical College, Fudan University, Shanghai, China

**Keywords:** music, middle cerebral artery occlusion, motor dysfunction, brain derived neurotrophic factor, glial fibrillary acidic protein

## Abstract

**Background/Aim:** Music-based therapy plays a role in central nervous system diseases. We aimed to explore the effect of different doses and durations of music therapy on motor function recovery after stroke and the underlying molecular mechanisms.

**Methods:** Adult male Sprague–Dawley rats were subjected to middle cerebral artery occlusion (MCAO) for 1 h, which was followed by reperfusion. In experiment 1, the rats that survived 1 week after MCAO surgery were randomly allocated into four groups (*n* = 10 per group): MCAO group, 1 h music group (Mozart K.448 music therapy 1 h per day for 2 weeks), 12 h music group (Mozart K.448 music therapy 12 h/day for 2 weeks), and accelerated music group (reversely accelerated music therapy 12 h for 2 weeks, AM group). In experiment 2, the survived rats were randomly divied into three groups: MCAO group, 12 h music group (music therapy 12 h/day for 3 weeks), and 12 h music-R group (music therapy 12 h/day for 2 weeks and rest for 1 week). Three neuroscores were evaluated daily, starting on the first day after surgery until the end of the experiment. The rats were killed 3 weeks after MCAO surgery in experiment 1 or 4 weeks after surgery in experiment 2. Nissl staining of infart core, peri-infarct zone, and motor cortex was performed to assess neuronal survival and regeneration. Western blot and immunofluorescence were used to detect the expression and distribution of brain-derived neurotrophic factor (BDNF) and glial fibrillary acidic protein (GFAP) in ipsilateral hemispheres.

**Results:** In the experiment of different music therapy doses, the motor function in the 12-h music group but not in the 1-h music group and AM group was significantly improved compared with that of the MCAO group. The BDNF protein level of the ipsilateral hemisphere motor cortex in the 12-h music group and the 1-h music group was higher than that of the MCAO group. The neurons and Nissl bodies were more in the 12-h music group than in the MCAO group. Immunofluorescence assay showed that a 12 h music therapy induces BDNF and GFAP accumulation at the damage boundary. In the experiment of different music therapy durations, 3 weeks music therapy (12 h music group) induced more longer cell synapses and more clearer cell-to-cell connections than 2 weeks music intervention (12 h music-R group). Moreover, the GFAP morphology in the 12-h music group was more similar to mature activated astrocytes than that in the 12-h music-R group.

**Conclusions:** Music therapy may improve poststroke motor function and promote neuronal repair in the long term. The mechanism may be through stimulating BDNF and GFAP secretion in the injured motor cortex.

## Highlights

- Music therapy can improve motor function after stroke.
- Music therapy induces BDNF accumulation in the motor cortex after stroke.
- Music therapy promotes neuronal repair and improves brain plasticity.

## Introduction

Stroke is a common and serious global health problem, and ischemic stroke accounts for ~75–85% of the total number of strokes (1). Stroke has become the second leading cause of death worldwide (2) and the first cause of mortality among Chinese urban and rural residents (3). Motor dysfunction due to stroke is one of the most common dysfunctions after stroke, accounting for about 70% of all cases, and seriously affects patients' activities (4). Currently, effective drugs for poststroke dysfunction are lacking (5–7). Although existing rehabilitation treatments based on exercise and occupational and physical therapies have greatly contributed to alleviating dysfunctions, they have extremely limited effects when the patient's disease course exceeds 1 year. Moreover, further improving patients' functional capacity is challenging, and the fatigue it brings to the patient also makes it difficult for the patient to persist in training.

Based on a practical function, music therapy is used to treat diseases or promote physical and mental health in accordance with systematic therapeutic procedures. As an emerging rehabilitation intervention, music therapy has achieved certain effects in various neurological disorders (8). Music therapy, including rhythmic auditory stimulation, can effectively improve poststroke motor function, such as upper limb function (9), gait, and stride length (10) and reduce reliance on compensatory movements (11). A previous study has shown that music therapy has different effects in regulating human blood pressure, heart rate, and hormones (12). In addition, music therapy can activate the auditory area, motor area, and hippocampus and plays a positive role in various neurological diseases (13, 14). Furthermore, music therapy has a positive effect on speech disorders, motor dysfunction, cognitive disorders, and mood disorders after stroke (8, 15).

Currently, Mozart's music is recognized as one of the most effective and therapeutic music to listen to. Studies have confirmed that it has beneficial effects in relaxing the body and mind and in regulating blood pressure, heart rate, and endocrine and cerebral blood flow (16, 17). In a rat model of epilepsy, brain-derived neurotrophic factor (BDNF) levels were increased after playing Mozart's music (16, 17). However, studies on the molecular mechanism of music therapy on stroke are few, and the optimal dose and duration of music therapy remains unclear.

Therefore, we aimed to explore the effect of music therapy on motor function recovery after stroke and to determine the influence of different doses and durations of music therapy on neuronal repair.

## Materials and Methods

### Animals

Adult male Sprague–Dawley rats weighing 220–250 g were kindly provided by Nantong University (Nantong, China). This study was approved by the Animal Care and Use Committee (IACUC) of the Affiliated Maternity and Child Health Care Hospital of Nantong University. All procedures were performed according to the guidelines issued by the IACUC (No. S20170422-112).

### Reagents and Antibodies

Primary antibodies BDNF (1:500), GFAP (1:2,500), and β-actin (1:10,000) used in this study were obtained from Millipore company (Billerica, MA, USA). Alexa Fluor^®^488- and Alexa Fluor^®^647-conjugated donkey anti-rabbit IgG were obtained from Jackson ImmunoResearch Laboratories (West Grove, PA, USA). The enhanced chemiluminescence (ECL) kit was from Thermo Scientifc (Rockford, IL, USA). Other chemicals were from Sigma (St. Louis, MO, USA).

### Middle Cerebral Artery Occlusion

All rats were raised for 1 week to acclimate to a temperature of 25°C and humidity of 55–75% and were exposed to a 12-h photoperiod (2,800 Lux). Rats were fed on water *ad libitum* and standard chow. In preparation for the middle cerebral artery occlusion (MCAO), 80 rats were continually anesthetized with 3% isoflurane, which was followed by intubation and ventilation with 2.0–2.5% isoflurane in a mixture of 30% oxygen and 70% air. They were inverted and fixed; subsequently, an area of the skin over the right carotid artery was exposed. After clipping the hair, the skin was scrubbed with betadine, rinsed in alcohol, and painted with iodine. A skin incision was made over the right common carotid artery. The internal and external carotid arteries were isolated and exposed. A 5–0 monofilament Nylon (Doccol Corporation; Sharon, MA, USA) was threaded into the internal carotid artery through a small incision on the external carotid artery. The suture was advanced toward the middle cerebral artery region to induce focal ischemia and was retained for 1 h; thereafter, it was removed for reperfusion. Sham rats underwent the same protocol without MCAO. Rats' body temperature was maintained with the heating pad and a warming blanket. Rats were weaned off the respirator until recumbent and alert. Ten sham-operated rats underwent identical surgery, but the suture was not inserted.

### Postoperative Care

Subcutaneous fluids were administered acutely (5 ms/kg sterile saline) following surgery to prevent dehydration. Rats were kept in a warm environment (e.g., 28°C) until fully conscious and were checked 2–3 h after surgery and at least once daily for 1 week. Postoperative pain was managed by intraperitoneal injection of 5 mg/kg ketoprofen. After 1 week, 10 of 80 MCAO rats died (the mortality rate is 12.5%).

### Music Intervention

#### Experiment 1

**Figure 1:**
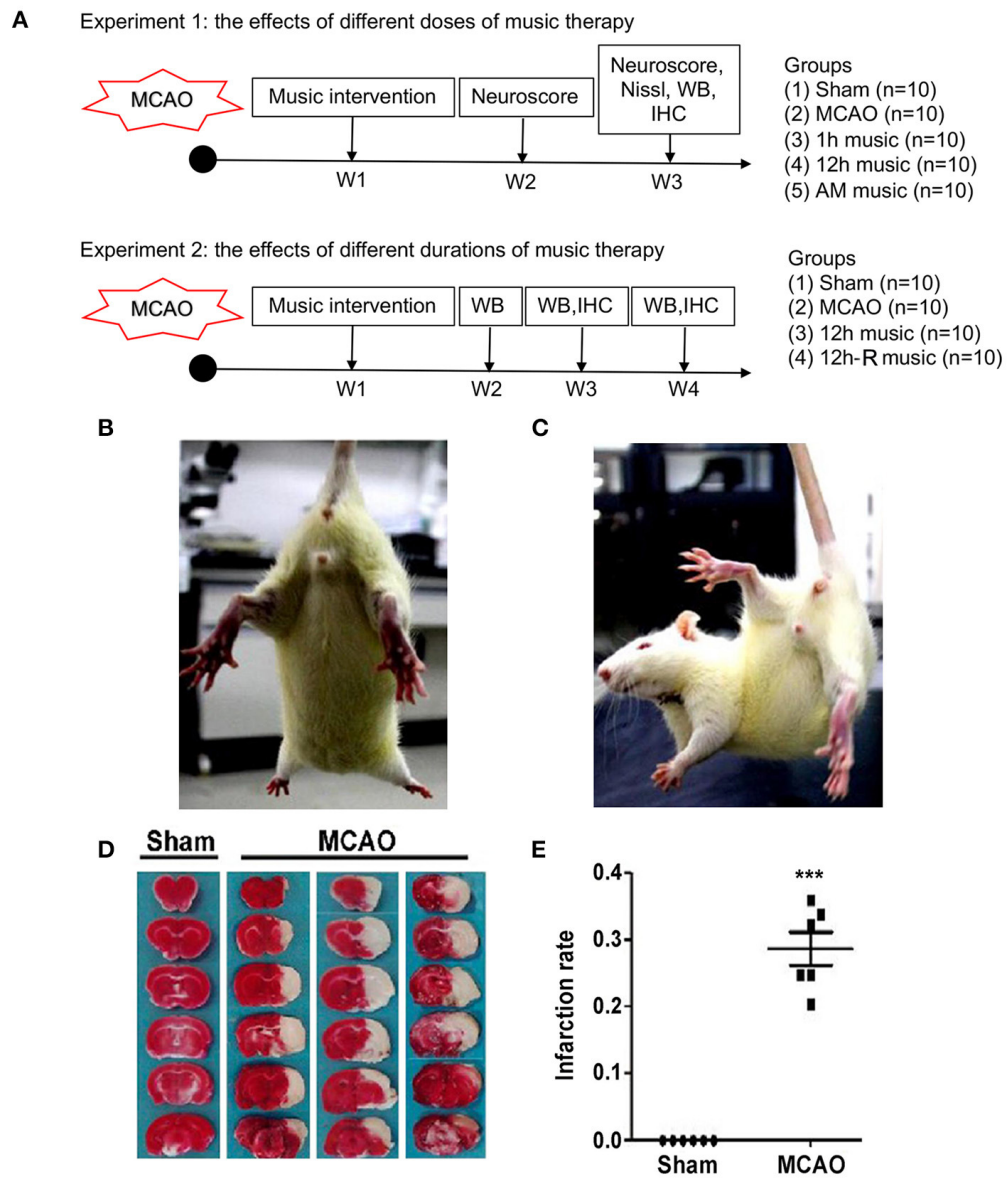
Experimental design and verification of cerebral MCAO surgery in rats. **(A)** Experimental design and animal groups. **(B,C)** The postural reflex test for sham rat and MCAO rat. **(D,E)** Coronal sections of the sham and MCAO rats were stained with TTC. AM, accelerated music; IHC, immunohistochemistry; MCAO, middle cerebral artery occlusion; W, week; WB, western blot. ***p < 0.001. The asterisks represent significant difference compared with the sham group.

To study the effects of different doses of music therapy, the survived rats suffered from MCAO surgery and sham-operated rats were divided into five groups (*n* = 10 per group): sham, MCAO, 1 h music, 12 h music, and accelerated music group (AM group). Music intervention started at 1 week after surgery. The rats in the 1-h music group were exposed to Mozart K.448 (65–75 dB) from 8 to 9 p.m. every day (17), those in the 12-h music group continually received the music therapy from 8 p. m. to 8 a.m., and those in the AM group were exposed to reversely accelerated music (8 × speedup). The music therapy continued for 2 weeks. The sham and MCAO groups were left undisturbed (~25 dB). The rats in both groups were housed at a density of one rat per cage (Figure 1A, upper panel).

#### Experiment 2

To study the effects of different durations of music therapy, the survived rats and sham-operated rats were randomly assigned to four groups (*n* = 10 per group): sham, MCAO, 12 h music (music without rest), and 12 h music-R (music with rest) group. At 1 week after operation, the rats in the 12-h music group continually received the music therapy for 3 weeks. Music intervention in the 12-h music-R group lasts for 2 weeks and then rest for 1 week (Figure 1A, lower panel).

### Neurobehavioral Evaluation

Modified neurological severity score (mNSS), general deficits, focal deficits, and beam walking tests were performed daily, starting on the first day after the surgery and were continued until the end of the experiment. The examiners were blinded to the procedures that the rat underwent. To evaluate the neuroprotective effects of music therapy, the original neurological severity score was modified (18). mNSS, which is a composite of motor, sense, beam balance, and reflection tests, was evaluated according to the scoring scheme presented in Table 1. The scoring of general deficits and focal deficits is presented in Tables 2, 3. To evaluate the motor function of the rats, postural reflex test was performed; the rats were gently lifted by the tail (1 m above the ground) and observed for body rotation and limbs flexing.

**Table 1:** The scoring scheme of the modified neurological severity score (mNSS) based on motor, sensor, beam balance and reflection tests.

**Test types**	**Points**
**Motor test**	
*Raising by the tail*	
Forelimb flexion	1
Hindlimb flexion	1
Head deflection more than 10 degrees on the vertical axis in 30 s	1
*Placing on the floor*	
Normal walk	1
Inability to walk	1
Turning to the sick side	2
Falling down to the sick side	3
**Sensor test**	
*Placing test*	
Vision and tactility	1
*Proprioceptive neuromuscular test*	
Push the rat paws to the edge of the table to stimulate limb	1
muscles	
**Beam balance test**	
Maintaining balance and stable posture	0
Holding on to the balance beam	1
Hugging the beam while one limb falling down from the beam	2
Hugging the beam while two limbs falling down, or spinning on	3
the beam	
Trying to maintain balance but falling down after 40 s	4
Trying to maintain balance but falling down after 20 s	5
Falling down without trying to stay steady within 20 s	6
**Reflection test**	
Head shaking when stimulating the external ear canal	1
Blinking when gently stimulating the cornea with cotton	1
Motor reflection to a brief noise from clapping	1
Seizures, myoclonus, or myospasm	1

**Table 2:** The scoring scheme of the general deficits in fur, ears, eyes, body position, spontaneous activity, and epilepsy.

**Test types**	**Points**
**Hair**	
Shiny, clean, and tidy	0
Vertical or dirty hair in 1–2 places usually around the nose and eyes	1
Vertical or dirty hair in more than 2 places	2
**Ears test**	
The ears are stretched to the side and back, and respond quickly to noise from the vertical direction	0
One or both ears are loose and weak, in a horizontal extension state, unable to extend back, and can respond to noise	1
One or both ears are loose and weak, in a state of lateral extension, unable to stretch back, and unable to respond to noise	2
**Eyes test**	
Eyes are open, clean and can react quickly	0
Eyes secrete watery or mucus-like discharge, and the eyes react slowly	1
Eyes are dark and dull	2
The cracks in the eyes are not round but oval and accompanied by discharge	3
Eyes are closed	4
**Posture**	
The rat can stand upright, its back is parallel to the ground, and when it is gently rocking, it can quickly maintain stability with its limbs	0
When the rat rests and walks, the back is bulged; the body is lowered when it is shaking instead of keeping the limbs stable	1
Head or part of the torso is resting on the ground	2
The rat's body is tilted to one side, but it can transform itself into an upright state	3
Inability to turn into upright and prone positions	4
**Spontaneous activity**	
Being alert, agile, and actively exploring	0
Being calm and quiet, start and stop exploring slowly and repeatedly	1
Listless, moving slowly, but no exploratory behaviors	2
Drowsy, unconscious, and hardly moving	3
No spontaneous movement, except for occasional responses to stimuli	4
**Epileptic behavior**	
Present with epilepsy symptoms	0
Suddenly rush out, repeatedly climb the cage wall or swing aimlessly	1
Be aggressive and nervous, look sluggish, or over-excited	3
Excessive excitement, galloping after being stimulated, showing localized seizures or generalized convulsions	9
Severe seizures with changes in breathing or consciousness	12

**Table 3:** The scoring scheme of the focal deficits in symmetry, gait, climbing, circling, and sensory response.

**Test types**	**Points**
**Symmetry test**	
Limbs are placed symmetrically under the body, with the tail straight back	0
The body is slight asymmetry and tilts to the affected side, the tail always deviates from the midline, and the limbs are not significantly asymmetry	1
The body is moderately asymmetry, the body is inclined to the affected side, the paralyzed limbs are stretched out, and the tail is off the midline	2
The body is significantly asymmetry, the body is bent into an arc, and the affected side is leaning on the table	3
The body is extremely asymmetrical, the body and tail are tightly bent, and the affected side is always on the table	4
**Gait test**	
Normal gait: flexible, symmetrical, fast	0
Mechanical gait: stiff, inflexible walking, moving in a hunched posture, or slowing down	1
Slight limp, asymmetry during grasping or movement	2
Severe limping, drifting, falling, obvious defects in gait	3
No spontaneous walking, no more than 3 steps after stimulation	4
**Climbing test**	
Quickly climb to the top edge of the slope	0
Climbing slowly, obviously tightening	1
Stay on the slope and do not slide down or climb up	2
Gradually decline, make efforts to stop the decline, but fail	3
Slid immediately and made no effort to stop it	4
**Circling test**	
Turn left or right equally	0
Spin mainly to one side	1
Turn sideways without continuity	2
Keep turning to one side	3
Rotating, swaying, moving slowly in circles, or maintaining motionless	4
**Sensory response test**	
Turn the head to the irritated area and stay away from the irritation	0
The affected side's reaction is delayed and weakened, while the contralateral reaction is normal	1
Absent response on the affected side, normal response on the contralateral side	2
Absent response on the affected side, weakened response on the contralateral side	3
Lack of bilateral proprioceptive response	4

### Triphenyl Tetrazolium Chloride Staining

The rats were decapitated under anesthesia at the end of the 24-h period after MCAO. Brain tissues were carefully obtained and washed in precold phosphate-buffered saline (PBS). The fresh brain tissues were immediately frozen at −20°C for 20 min and subsequently cut into 2-mm-thick coronary sections. The coronal sections were incubated in 2% triphenyl tetrazolium chloride (TTC) (Sigma-Aldrich, St. Louis, MO, USA) prepared in PBS at 37°C in the dark for 20 min. The coronal sections were then fixed in 4% paraformaldehyde overnight. The infarction rate was examined using Image J software (NIH, Bethesda, MD, USA).

### Nissl Staining

Tissue sections on coated slides were washed for 10 min three times in PBS. Thereafter, the slides were immersed in chloroform for 30 min and subsequently in acetone for 15 min. The tissues were rehydrated in graded ethyl alcohol (100, 95, and 70%; 30 s each). The slides were rinsed in distilled water twice and stained using cresyl violet (Beyotime, Shanghai, China) for 15–30 min at room temperature. Moreover, the slides were washed in distilled water, differentiated in ethyl alcohol (70, 95, and 100%; 30 s each), immersed in chloroform for 5 min, and incubated with 95% ethyl alcohol (the pH was adjusted to 4.1 using acetic acid). Thereafter, the slides were treated with ethyl alcohol (95, 100, and 100%; 30 s each), which was followed by deparaffinization with xylene (three times for 2–3 min each treatment). Lastly, the slides were mounted in neutral balsam.

### Western Blot Analysis

The tissues were collected from the right motor cortex and lysed in radio-immunoprecipitation assay lysis buffer (Beyotime) supplemented with phenylmethanesulfonyl fluoride (Beyotime) and protease inhibitor cocktail (Roche, Mannheim, Germany). After homogenate extraction, the extract was maintained on ice for 30 min. The supernatant was collected by centrifugation (4°C, 12,000 rpm, 15 min), which was performed twice. Protein concentration was examined using bicinchoninic acid kit (Thermo Science Fisher, Waltham, MA, USA) according to the user's manual. Approximately 20 μg of proteins were loaded and electrophoresed on 10% sodium dodecyl sulfate-polyacrylamide gel electrophoresis. The proteins were electronically transferred onto a polyvinylidene fluoride membrane (Millipore, Bedford, MA, USA). The membrane carrying the proteins was blocked with 5% skim milk for 90 min at room temperature. After washing in Tris-buffered saline–Tween (TBST), the membrane was incubated with primary antibodies against BDNF, GFAP, and β-actin (Millipore, Billerica, MA, USA) overnight at 4°C. The membrane was rinsed in TBST for 15 min three times; subsequently, it was incubated with horseradish peroxidase-conjugated goat-anti-mouse or goat-anti-rabbit IgG (Bioworld, Minneapolis, MN, USA) at 37°C for 1 h. Protein bands were determined using a chemiluminescent substrate kit (Thermo Science Fisher) before visualization using Image J software.

### Immunohistofluorescence

The frozen brain tissue section was washed in PBS for 15 min three times and inubated with 1% Triton in PBS for 15 min. Slides were inucbated with 5% normal donkey serum for 90 min. Thereafter, the slides were incubated with primary antibodies against BDNF and GFAP at 4°C overnight and were subsequently washed in PBS for 20 min three times. Alexa Fluor^®^488- and Alexa Fluor^®^647-conjugated donkey-anti-rabbit IgG were used to amplify the protein signaling (Jackson ImmunoResearch, West Grove, PA, USA), and the incuabtion was performed at room temperature for 60 min. To detect nuclei, 2-(4-amidinophenyl)-6-indolecarbamidine dihydrochloride (Beyotime) was incubated with the slides for 15 min. The slides were photographed with a confocal scanning microscope (Leica, Wetzlar, Germany) installed with Image J software.

### Statistical Analysis

Statistical analysis was conducted with IBM SPSS Statistics 21 software (IBM Corporation, Armonk, NY, USA). Data are presented as menas ± standard error mean of at least three independent experiments. One- or two-way analysis of variance was used to compare the difference between multiple groups. Least significant difference was used for pairwise comparison; *U*-test, for non-parametric comparision. A difference with *p* < 0.05 was considered to be statistically significant.

## Results

### Effects of Music on Neurobehavioral Outcomes of MCAO Rats

Postural reflex test was performed after MCAO. Sham rats showed no obvious neurological deficit during limb extension (Figure 1B), while the MCAO rats flexed to the affected side, retracted and internally rotated the left limb, and tilted the trunk to the affected side (Figure 1C). Moreover, TTC staining showed considerable infarct changes in the MCAO group compared with the sham group (*p* < 0.001) (Figures 1D,E). These results indicated that MCAO model in rats was effectively established.

**Figure 2:**
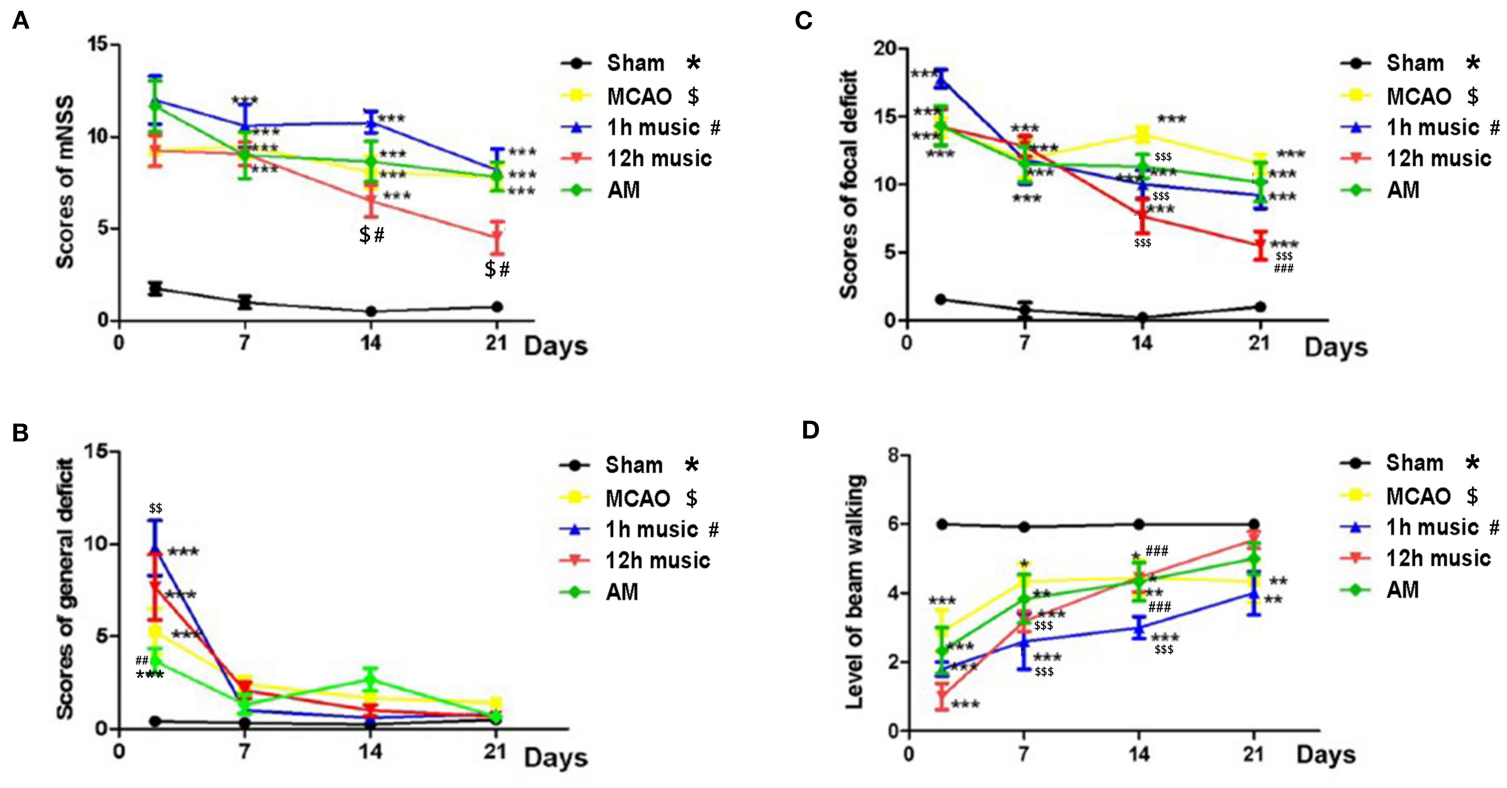
Effects of music on neurobehavioral outcomes of MCAO rats. **(A)** mNSS, **(B)** general deficit scores, **(C)** focal deficit scores, and **(D)** beam walking level of the sham, MCAO, 1 h music, 12 h music, and AM rats. Music therapy was initiated on the eighth day after MCAO. *^, $, #^*p* < 0.05; **^, $$, ##^*p* < 0.01; ***^, $$$, ###^*p* < 0.001. The asterisk, dollar sign, and number symbol represent a significant difference compared with the sham, MCAO, and 1 h music groups, respectively.

Music intervention was initiated on day 8 post-MCAO and was provided for 2 weeks. We assessed the effects of music therapy on neurological function. The mNSS of the sham group was significantly lower than that of the MCAO, 1 h music, 12 h music, and AM groups on days 2, 7, 14, and 21 post-MCAO (*p* < 0.001) (Figure 2A). Before music intervention, mNSS showed no significant difference between MCAO and 1 h music, 12 h music, or AM groups (*p* > 0.05) (days 2 and 7 post-MCAO). The 12-h music group showed a significantly decreased mNSS compared with the MCAO group on days 14 and 21 post-MCAO (*p* < 0.05) (Figure 2A). The mNSS of the 12-h music group was lower than that of the 1-h music group on days 14 and 21 (*p* < 0.05) (Figure 2A). Moreover, MCAO significantly caused general deficits addressing the hair, ears, eyes, posture, spontaneous activity, and epileptic behavior on the second day after MCAO (*p* < 0.01 or *p* < 0.001) (Figure 2B). MCAO resulted in focal deficits affecting symmetry, gait, climbing, circling, and sensory response (*p* < 0.05). Music therapy significantly ameliorated the focal neurological deficits in the 12-h music group on days 14 and 21 post-MCAO (*p* < 0.001) (Figure 2C). In addition, the beam walking ability, which was impaired by MCAO, improved in the 12-h music group (*p* < 0.001) (Figure 2D).

### Effect of Music Therapy on Brain Tissue Loss and Neuronal Repair in MCAO Rats

**Figure 3:**
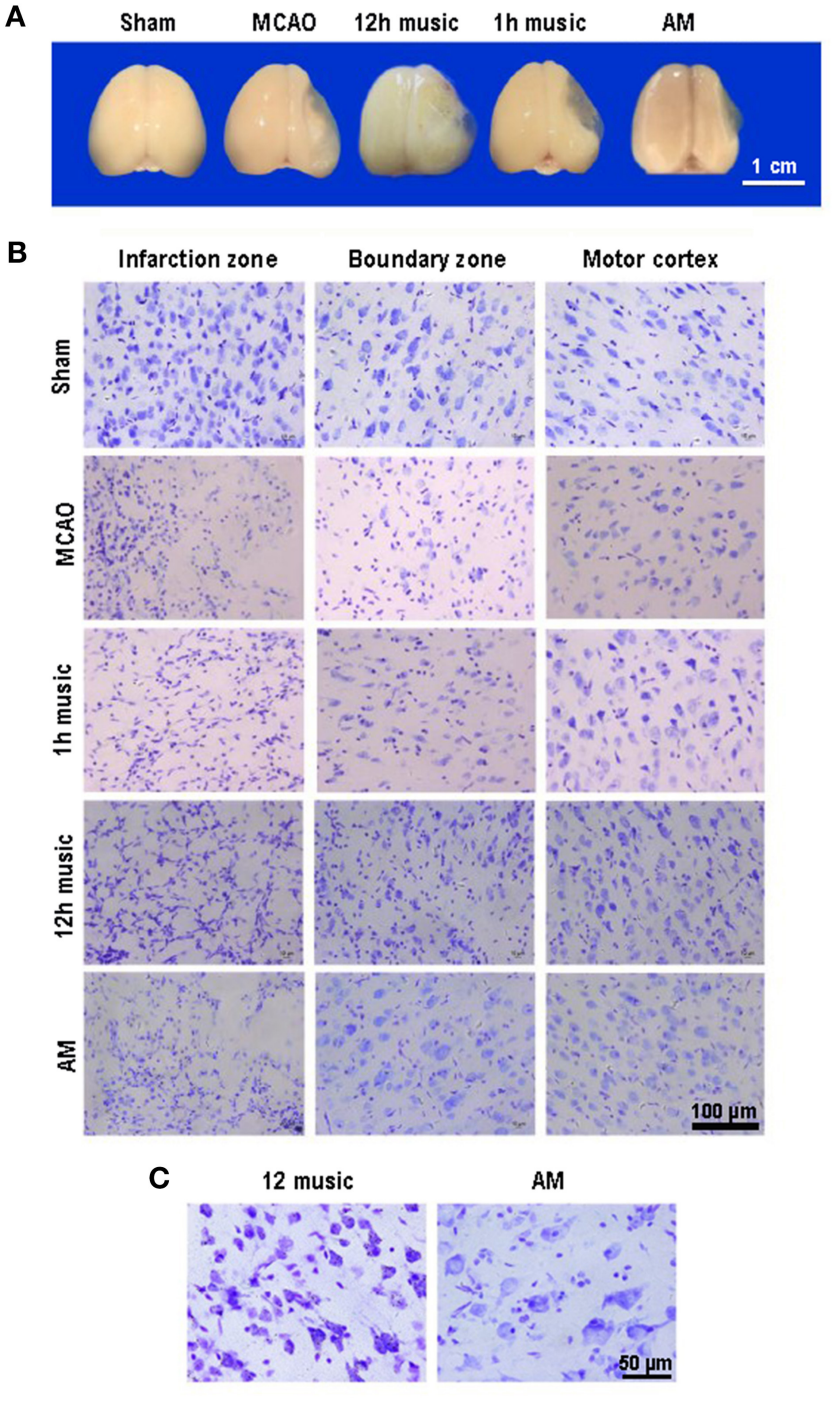
Music therapy induced changes in brain morphology and neuronal repair. **(A)** Brains of sham, MCAO, 1 h music, 12 h music, and AM rats 21 days after MCAO operation. **(B)** Comparison of the effects of forward music and those of 8 × speedup reverse music on neuron repair. Music therapy was started on the eighth day after MCAO, and the treatment continued for 2 weeks. **(C)** Enlarged morphology comparison between 12 h music group and AM group.

The MCAO rats exhibited brain atrophy and liquefied changes. In the 12-h music group, brain atrophy was improved (Figure 3A). Furthermore, we performed Nissl staining of the infarct area, boundary, and motor cortex in the sham, MCAO, 1 h music, 12 h music, and AM groups. After 2 weeks of music intervention, the number of nerve cells was increased, the intercellular space reduced, and the neurons and Nissl bodies in infarction, boundary, and motor cortex increased in the 1-h music and 12-h music groups compared with the MCAO group. Particularly, the 12-h music therapy increased nerve cell generation and induced network structure formation between nerve cells (Figure 3B). The normal neurons and Nissl bodies around the injured area was higher in the 12-h music group than in the AM group; the AM group was dominated by vacuole-like cells instead of Nissl bodies (Figure 3C). Thus, in MCAO, which could result in significant damages to the brain structures, a 12-h music therapy has a therapeutic value.

### Effects of Music Therapy on BDNF and GFAP Expression

**Figure 4:**
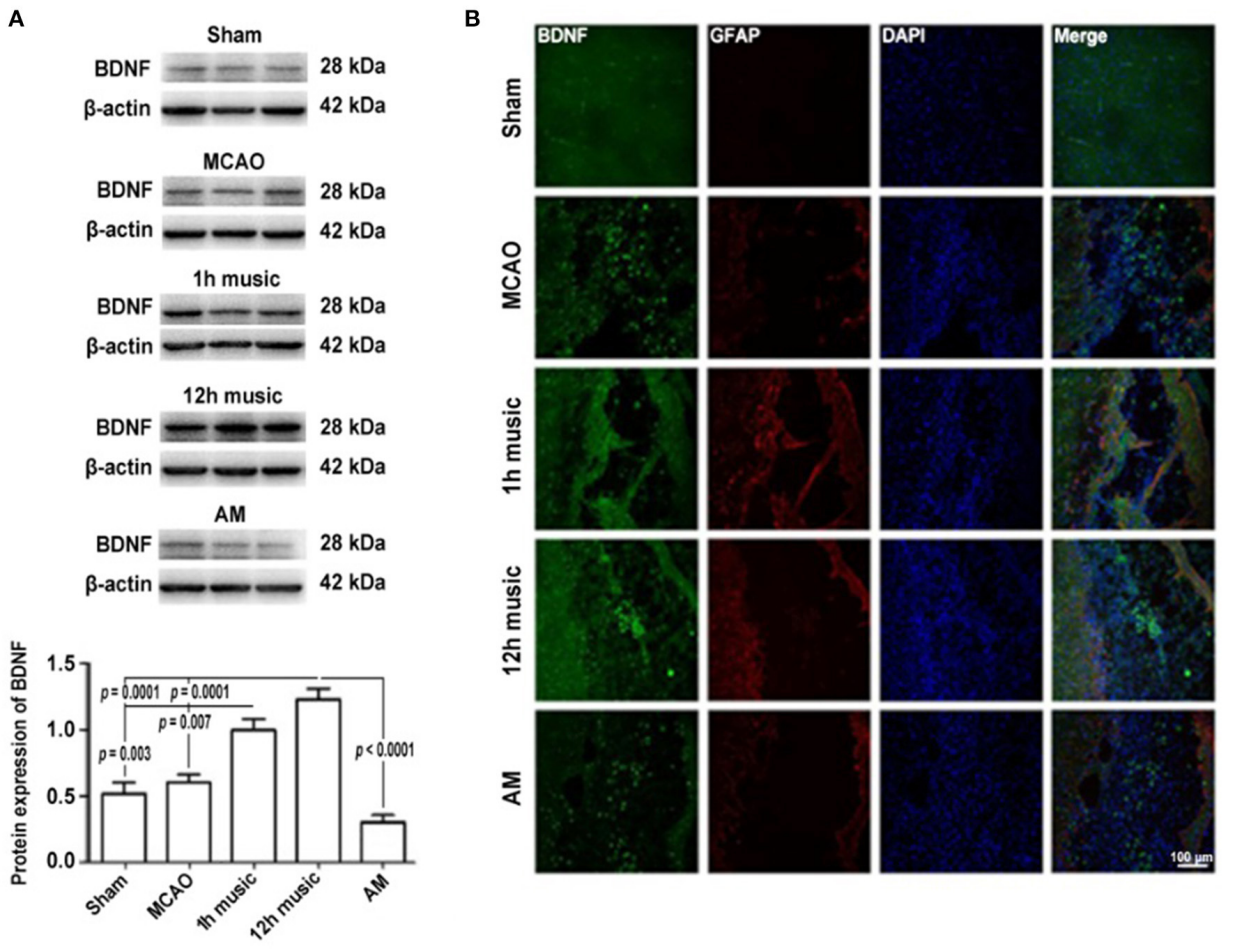
Music therapy after MCAO increased the BDNF expression. **(A)** Western blot analysis of BDNF in the lesioned area. **(B)** Relative fluorescence intensity of BDNF and GFAP in the motor cortex. Music therapy was started on the eighth day after MCAO, and the treatment continued for 2 weeks.

To explore the molecular mechanisms in the amelioration of cerebral ischemia–reperfusion injury by music therapy, the BDNF level in the motor cortex was examined. After 2 weeks of music intervention, the BDNF content in the right motor area was significantly higher in the 12-h music group than that in the sham (*p* = 0.0001), MCAO (*p* = 0.0001), or AM (*p* < 0.0001) groups (Figure 4A). No significant difference in BDNF content between the MCAO group and AM group was found (*p* > 0.05). Similarly, the BDNF content was significantly higher in the 1-h music group than that in the sham (*p* = 0.003) or MCAO groups (*p* = 0.007). However, no significant difference in BDNF content between the 1-h music and 12-h music groups was observed (*p* > 0.05).

To better understand the repair of the infarct area, we performed immunofluorescence analysis to detect the expression and distribution of BDNF and GFAP after 2 weeks of music intervention. BDNF and GFAP in the sham, MCAO, 1 h music, 12 h music, and AM groups were evaluated; the 12-h music group showed the highest content and the widest distribution of BDNF in new undifferentiated cells and astrocytes (Figure 4B). The BDNF fluorescence intensity in the 1-h music group was slightly lower than that in the 12-h music group, although the GFAP content was relatively high and the connection was formed in the liquefaction zone in the 1-h music group. Relatively few newly born and undifferentiated cells were observed in the 1-h music group. In the MCAO group, BDNF distribution was scattered and BDNF was detected in newly born and undifferentiated cells. GFAP fluorescence intensity was weak and GFAP was distributed in dots. In the AM group, BDNF was distributed in dots, and the BDNF fluorescence intensity was not significantly different from that in the MCAO group; the nuclei were evenly distributed in the liquefaction zone, and low GFAP expression was observed.

### Persistent Effects of 12 h Music Therapy on BDNF and GFAP Expression

**Figure 5:**
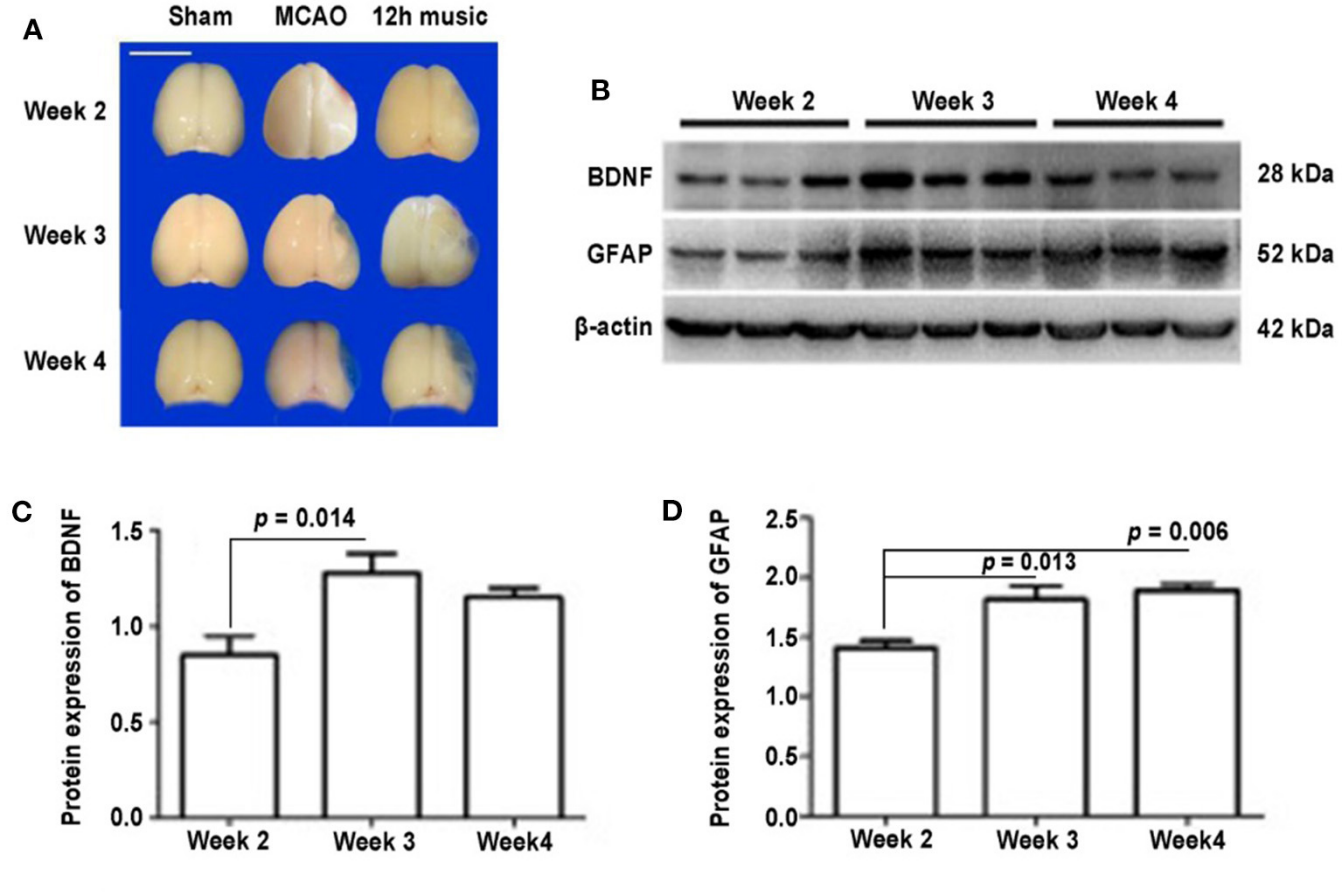
Effects of 12 h music therapy on brain morphology and the expression of BDNF and GFAP at 2, 3, and 4 weeks after MCAO. **(A)** Brains of sham, MCAO, and 12 h music groups at 2, 3, and 4 weeks after MCAO. Bar, 1 cm. **(B)** Western blot analysis of BDNF and GFAP in the lesioned area of the 12-h music group. Quantification of **(C)** BDNF and **(D)** GFAP examined by Western blot.

To determine the duration of the effects of one course of 12 h music therapy, we examined the expression and distribution of BDNF and GFAP at 1, 2, and 3 weeks after the music intervention, which corresponded to the second, third, and fourth week after MCAO. As shown in Figure 5A, the brains of the MCAO group gradually liquefied and collapsed and were not filled with new jelly until the fourth week after the operation. One week after the music intervention, the brains of the 12-h music group were intact and full, and the infarct core area was semipermeable; 2 weeks after the intervention, the non-infarcted area was filled with transparent jelly, and filiform connections were visible; and 3 weeks after the intervention, the brain shape remained full and brain volume was similar to that of the sham group.

**Figure 6:**
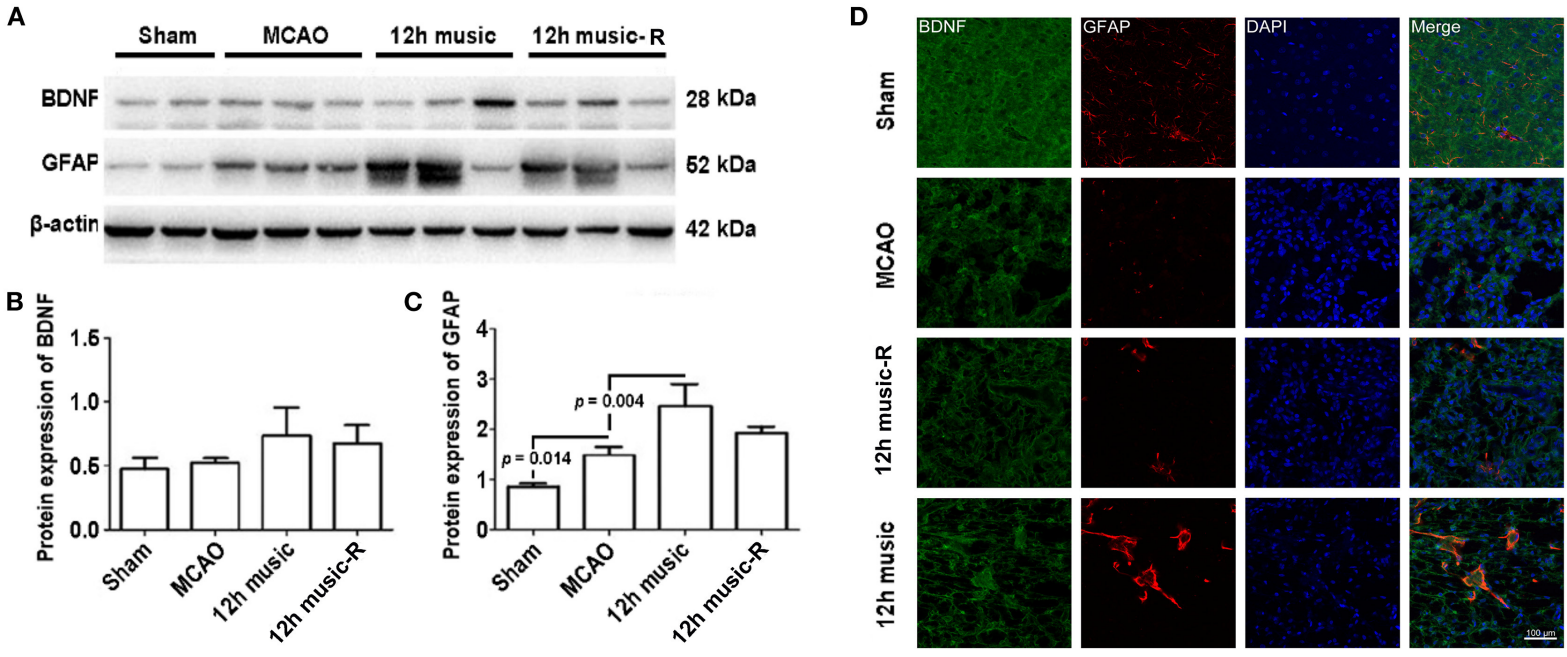
Effects of 12 h music therapy on BDNF and GFAP expressions at 4 weeks after MCAO. **(A)** Western blot analysis of BDNF in the lesioned area. Quantification of **(B)** BDNF and **(C)** GFAP examined by Western blot. **(D)** Relative fluorescence intensity of BDNF and GFAP in motor cortex. The BDNF and GFAP expressions were examined after 3 weeks of intervention in the 12-h music group and after 2 weeks of intervention plus 1-week rest in the 12-h music-R group.

No significant difference in BDNF content among the sham, MCAO, and 12 h music groups was found after 1 week of music intervention. The BDNF content in the 12-h music group was significantly higher than that of the MCAO and sham groups after 2 weeks of intervention (*p* = 0.014). However, no significant difference was observed among the three groups after 3 weeks of intervention (*p* > 0.05) (Figures 5B,C). The GFAP content in the 12-h music group was significantly higher after 3 weeks of intervention than that after 1 week of intervention. However, no significant difference in GFAP content in the 12-h music group was found between 3 and 2 week interventions (*p* > 0.05) (Figures 5B,D). Moreover, the BDNF content in the 12-h music-R group (in which music intervention was performed for 2 weeks followed by 1 week of rest) was not significantly different from that in the sham, MCAO, and 12 h music groups (*p* > 0.05) (Figures 6A,B). Similarly, no statistically significant difference in the GFAP content was noted between the 12-h music and the 12-h music-R groups (Figures 6A,C). Additionally, immunofluorescence assay showed BDNF accumulation in newborn cells and astrocyte cell morphology in the music group, and the GFAP fluorescence intensity was stronger in the music group than that in the MCAO group (Figure 6D).

**Figure 7:**
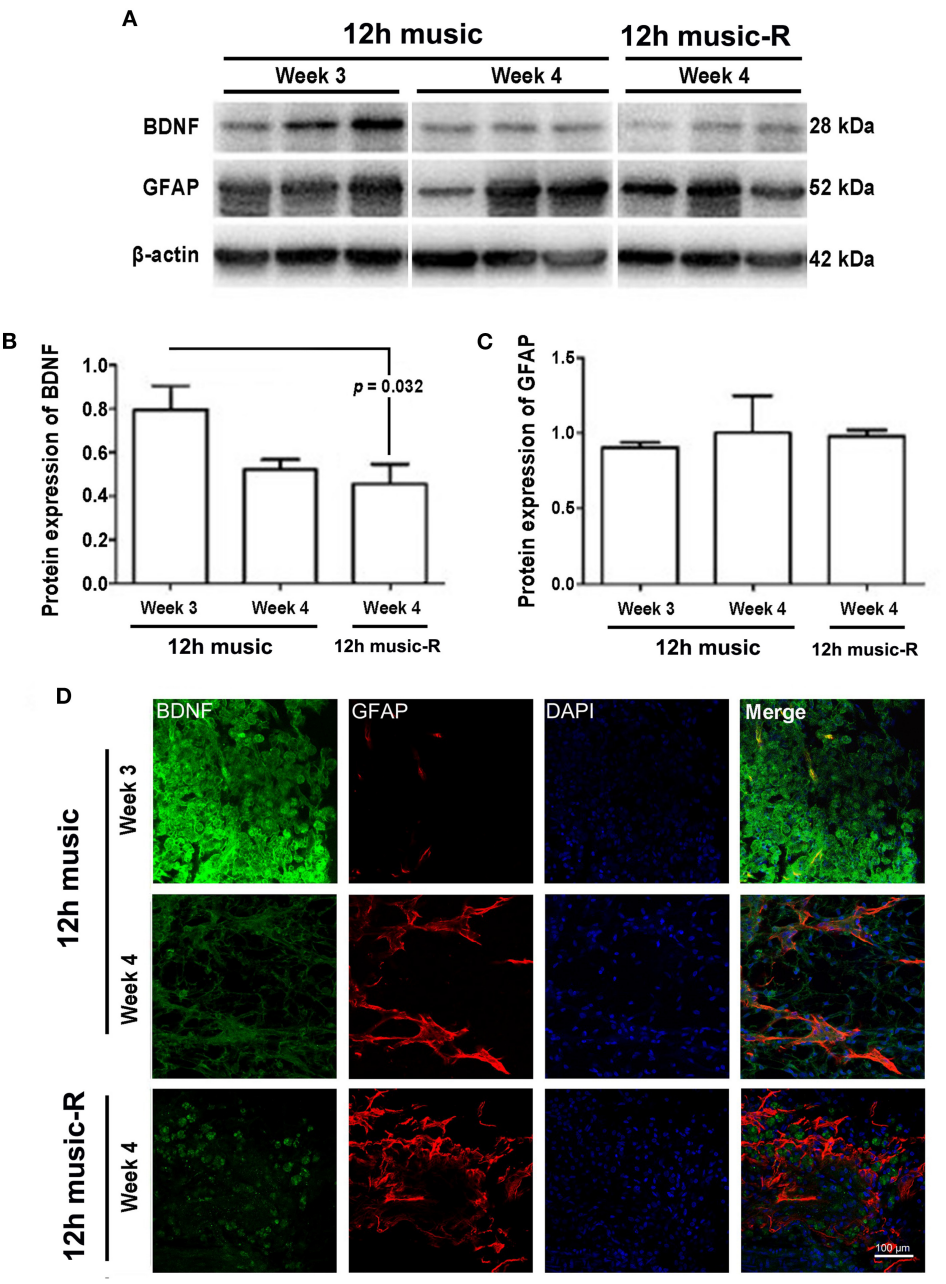
Persistent effects of 12 h music therapy on BDNF and GFAP expression. **(A)** Western blot analysis of BDNF in the lesioned area. Quantification of **(B)** BDNF and **(C)** GFAP examined by Western blot. **(D)** Relative fluorescence intensity of BDNF and GFAP in the motor cortex. Rats in the 12-h music group continually received the intervention for 3 weeks; those in the 12-h music-R group, for 2 weeks plus 1-week rest. BDNF and GFAP levels were examined at 3 and 4 weeks after MCAO.

The different courses of music intervention resulted in increased BDNF content in the motor area, which subsequently decreased. Music intervention lasts for 2 weeks and then rest for 1 week; the BDNF level was significantly lower in the 12-h music-R group than that of the music group with 2-week intervention (*p* = 0.032) (Figures 7A,B). Although the GFAP levels in the 12-h music and 12-h music-R groups were significantly different after 2 weeks of intervention, the difference was not statistically significantly after 3 weeks of intervention (*p* > 0.05) (Figures 7A,C). No significant difference in BDNF fluorescence intensity was found between the 12-h music and the 12-h music-R groups (Figure 7D). Moreover, the cells in the 12-h music and 12-h music-R groups gradually differentiated into mature astrocytes; both groups showed better cell differentiation than the MCAO group. In the MCAO group, the BDNF in the newborn cells of the infarct core area formed a network structure; however, the cell boundaries were unclear, clear synaptic connections are lacking, and GFAP was mostly distributed in dots. In the 12-h music-R group, the newborn cells had short synaptic connections, which form an astrocyte morphology. In the 12-h music group, the continuous intervention resulted in longer synapses and clearer cell-to-cell connections, and the GFAP morphology was closer to that of mature activated astrocytes, and the cells differentiated well.

## Discussion

Motor dysfunction, which directly affects a patient's activities of daily living, is the most common dysfunction after stroke and is related to patient's quality of life (19). A number of clinical studies have confirmed that the use of music rehabilitation measures can significantly improve poststroke dysfunction, including motor dysfunction (20) and speech disorder (21). Thus, this study aimed to focus on the effect of music on poststroke movement disorders. Here, an MCAO rat model was used to simulate the pathological state of human stroke, and music therapy was used as the intervention.

Music can regulate various human body activities. A previous study has shown that hypertensive rats that listen to Mozart's music for 2 h a day under light conditions showed reduced heart rate without blood pressure changes and that Ligeti's music can increase blood pressure (22). Studies have also shown that music can regulate breathing and cardiovascular contraction and relaxation and has a role in the inflammatory response regulation (23). For example, Mozart's piano sonatas has been proven to reduce the expression of inflammation markers and improve the activation of the immune system natural killer cells (23).

Our study focused on the influence of music on the MCAO and did not involve a discussion on specific musical elements. Given that the therapeutic effects of different musical elements vary, our study used Mozart K.448, which has been frequently studied. However, our study adopted the form of three movements, which was played in a loop; more musical elements were incorporated to make the rhythm, melody, and chord more diverse, which may be helpful to achieve a more comprehensive stimulation effect.

In our study, a 12-h music therapy ameliorated focal neurological deficits due to MCAO, suggesting that the music therapy can effectively improve poststroke motor dysfunction. A previous clinical research focused on active music therapy, that is, active training based on musical elements (24). In our study, passive music therapy was used as the intervention; however, the activities of the rats were not restricted during the intervention period to simulate clinical completion of corresponding training based on music. Thus, our results are more reliable. Previous studies proposed that different musical elements including rhythm, melody, pitch, and musical cycle, play a role in the improvement of physiological dysfunctions (25–27). Rhythm-based music therapy, such as auditory rhythmic stimulation, has a significant effect on improving the patient's walking function (28), and participatory music therapy has a significant effect on improving upper limb function (29). Additionally, it has been shown that music therapy activates the limbic system, induces the production of neuropeptides associated with happiness and reward mechanisms, and continually regulates mood (30).

BDNF, which is an essential neurotrophic factor, plays an important role in promoting neuron growth by binding to the high-affinity tyrosine kinase receptor β (31). Music can stimulate pathological or healthy mice to release BDNF in different areas of their brains, especially in the hippocampus CA3, dentate gyrus, prefrontal cortex, amygdala, and hypothalamus (32, 33). A previous study confirmed that exogenous BDNF effectively repairs the ischemic penumbra and protects the residual neurons against MCAO *in vivo* (34). Our results suggested that music therapy promotes BDNF accumulation and influences neuron repairing and synapse regeneration, whereas noise has an adverse effect on brain repair after stroke. In addition, our results showed that music therapy induces GFAP distribution in the brain. GFAP, which is an astrocyte-specific intermediate filament component, plays a role in the improvement of ischemic brain damage due to focal cerebral ischemia with partial reperfusion (35). Moreover, music therapy promotes cell regeneration in the early stage and subsequently induces the differentiation and growth of synapses.

Music therapy may have a persistent effect on brain plasticity. Studies demonstrated that combined music exercise can continually reduce the loss of white matter and gray matter in the brain of the elderly individuals (36). Another study showed that the volume of gray matter and white matter is increased in people who received music training for a long time (37, 38). The use of musical instruments expands the connection fibers between the auditory and motor zones, thereby further enhancing the connection between the remote areas and such connection will not disappear with time (8). However, previous studies reported that the long-term effects of music therapy on some degenerative neuropathy are unsatisfactory. A study showed that with the cessation of music therapy, the motor function of Parkinson's patients cannot be maintained (39). In our study, although the BDNF content decreased in the fourth week, the cells in the injured area were well-differentiated and no significant difference between the 12-h music and 12-h music-R groups were found. Hence, music therapy has a long-term effect on brain plasticity.

## Conclusions

In summary, an appropriate dose of music therapy can effectively alleviate poststroke motor dysfunction, stimulate BDNF and GFAP secretion in the injured motor cortex, promote cell regeneration in the core area of the infarction, and induce the repair of residual neurons in the peripheral area. The mechanism may be through stimulating the absorption and resecretion of BDNF and GFAP by astrocytes, which in turn regulate the redistribution of BDNF and GFAP in time and space.

## Data Availability Statement

The raw data supporting the conclusions of this article will be made available by the authors, without undue reservation.

## Ethics Statement

The animal study was reviewed and approved by Animal Care and Use Committee (IACUC) of Affiliated Maternity and Child Health Care Hospital of Nantong University.

## Author Contributions

All authors listed have made a substantial, direct and intellectual contribution to the work, and approved it for publication.

## Conflict of Interest

The authors declare that the research was conducted in the absence of any commercial or financial relationships that could be construed as a potential conflict of interest.
